# Unidirectional fluxes of monovalent ions in human erythrocytes compared with lymphoid U937 cells: Transient processes after stopping the sodium pump and in response to osmotic challenge

**DOI:** 10.1371/journal.pone.0285185

**Published:** 2023-05-04

**Authors:** Valentina E. Yurinskaya, Alexey V. Moshkov, Irina I. Marakhova, Alexey A. Vereninov

**Affiliations:** Institute of Cytology, Russian Academy of Sciences, St-Petersburg, Russia; Griffith University Menzies Health Institute Queensland, AUSTRALIA

## Abstract

Recently, we have developed software that allows, using a minimum of required experimental data, to find the characteristics of ion homeostasis and a list of all unidirectional fluxes of monovalent ions through the main pathways in the cell membrane both in a balanced state and during the transient processes. Our approach has been successfully validated in human proliferating lymphoid U937 cells during transient processes after stopping the Na/K pump by ouabain and for staurosporine-induced apoptosis. In present study, we used this approach to find the characteristics of ion homeostasis and the monovalent ion fluxes through the cell membrane of human erythrocytes in a resting state and during the transient processes after stopping the Na/K pump with ouabain and in response to osmotic challenge. Due to their physiological significance, erythrocytes remain the object of numerous studies, both experimental and computational methods. Calculations showed that, under physiological conditions, the K^+^ fluxes through electrodiffusion channels in the entire erythrocyte ion balance is small compared to the fluxes through the Na/K pump and cation–chloride cotransporters. The proposed computer program well predicts the dynamics of the erythrocyte ion balance disorders after stopping the Na/K pump with ouabain. In full accordance with predictions, transient processes in human erythrocytes are much slower than in proliferating cells such as lymphoid U937 cells. Comparison of real changes in the distribution of monovalent ions under osmotic challenge with the calculated ones indicates a change in the parameters of the ion transport pathways through the plasma membrane of erythrocytes in this case. The proposed approach may be useful in studying the mechanisms of various erythrocyte dysfunctions.

## Introduction

Rapidly advancing genomics, transcriptomics, and proteomics have overshadowed cell physiology, which focuses on the cell as a whole. However, we believe that a holistic view of cellular ionic homeostasis is still relevant. The tight interdependence of ion fluxes across the plasma membrane makes it difficult or even impossible their assessment by direct measurement. Computational methods help to analyze the mechanisms of ion transport through the cell membrane. The first integrated mathematical model of human erythrocytes was developed by Lew and Bookchin [1]. A new JAVA version of a model on the homeostasis of human red blood cells (RBCs) was applied to investigate the changes that erythrocytes undergo during single capillary transits [2].

The previous mathematical descriptions was focused only on certain cells types, such as human red blood cells and reticulocytes [1–5], guinea-pig cardiomyocytes [6], or frog skeletal muscle [7,8] and included parameters whose experimental determination can be challenging. Recently, we have developed a simpler and a more universal computer program that allows, based on the minimum required experimental data, to find all the characteristics of ion homeostasis and a list of all unidirectional fluxes of monovalent ions through the main pathways in the cell membrane both in the stationary state and during the transient processes. The flux equations in our computation are similar to those used in the fundamental works of Jakobsson [9] and Lew [1,3].

Our approach is based on the use of the thermodynamic classification of ion transport systems through the cell membrane and does not depend on the mechanism of ion movement. It takes into account all the main types of ion-conducting pathways through the plasma membrane: sodium pump, electrically conductive channels, and all main types of cation-chloride cotransporters [10–15]. Until now, the reliability of the proposed description has been successfully shown for transient processes such as Na/K pump blockage by ouabain or replacing extracellular Na^+^ with Li^+^, as well as in staurosporine-induced apoptosis in proliferating lymphoid U937 cells [10–14].

The main goal of the current work was to validate the suitability of our computer program for human erythrocytes, whose ion transport through the membrane is much slower compared to proliferating cells. Erythrocytes, due to their structural simplicity, have been a popular object of study for cell physiologists for many decades [1,3,16–23, see also reviews 24–27]. They are non-nucleated cells, lacking all intracellular organelles, and they can be considered as proper model systems for studying the transport of ions across the plasma membrane. The main function of erythrocytes is the transport of gases, their active participation in blood coagulation has also been known for a long time. Defects in the properties of erythrocytes and disorders of their ionic and water homeostasis are associated with various diseases: sickle cell anemia [28], hereditary spherocytosis [29], Gardos channelopathy [30], peripheral vascular disease [31], etc. Erythrocytes have recently been shown to cause vascular dysfunction in COVID-19 [32]. Despite years of research, RBCs remain “a mysterious and fascinating study objects” [33]. Recently, mainly as a result of researches focused on the disease, there has been a significant increase in the amount of data on the presence and activity of signaling pathways that regulate the function of erythrocytes [34–37].

The present study shows that our proposed mathematical description predicts well changes in cellular ionic balance caused by sodium pump blockage in human erythrocytes, as was the case for proliferating lymphoid U937 cells, and can also be useful in elucidating which ion pathway may be responsible for specific changes in erythrocyte homeostasis caused by reasons other than stopping the sodium pump. The developed computer program makes it possible to determine which changes in the properties of the ion transport pathways will lead to the coincidence of the calculated and experimental data, and thus outline the ways for further research.

## Materials and methods

### Reagents

Hank’s saline solution (HSS), RPMI 1640 medium, fetal bovine serum (FBS, HyClone Standard), and gentamycin were purchased from Biolot (Russia). Ouabain (Oua) was from Sigma-Aldrich (Germany), Percoll was purchased from Pharmacia (Sweden). Salts and sucrose were of analytical grade and were from Reachem (Russia).

### Cells and solutions

The procedures involving human cells were performed in accordance with the standards of the Declaration of Helsinki (1989) and approved by the Institute of Cytology Ethics Committee. Written informed consent was obtained from all donors who provided tissue. RBCs were obtained from fresh blood from the fingers of 4 adult volunteer donors in our laboratory. Fresh blood was transferred at a dilution of approximately 1:50 into HSS (containing in mM: NaCl 140, KCl 5, CaCl_2_ 1, MgCl_2_ 0.4, MgSO_4_ 0.5, glucose 6) supplemented with 10 μg/ml gentamycin at 37°C. Only freshly isolated erythrocytes were used. RBCs were incubated in the medium with or without addition of 30 μM ouabain for the 0–48 h or for 2 h in medium with different osmolarity at 37°C. Hypoosmolar medium (200 mOsm) was obtained by diluting the standard HSS medium with distilled water in a ratio of 2:1. To reduce the concentration of salts in the medium without changing its osmolarity HSS was diluted with an iso-osmotic sucrose solution in a ratio of 2:1. To prepare a hyperosmolar medium 100 mM NaCl or 200 mM sucrose was added to standard HSS. The osmolarity of solutions was checked with the Micro-osmometer Model 3320 (Advanced Instruments, USA). During the experiments, the glucose content in the medium did not change significantly.

Human histiocytic lymphoma U937 cells were obtained from the shared research facility “Vertebrate cell culture collection” supported by the Ministry of Science and Higher Education of the Russian Federation (Agreement № 075-15-2021-683). U937 cells were cultured in RPMI 1640 medium supplemented with 10% FBS at 37°C and 5% CO_2_ and treated with 10 μM ouabain.

### Determination of cell ion, water and haemoglobin contents

RBCs were pelleted by centrifugation in an Eppendorf (Minispin plus) centrifuge until a centrifuge speed of 10,000 rpm was reached (6,700 x g), and washed three times with MgCl_2_ solution (96 mM), each time centrifuging as before. RBCs were lysed with distilled water. The erythrocytes were not treated with trichloroacetic acid (TCA), as in the case of U937 cells, since the TCA supernatant cannot be used for the spectrophotometric determination of haemoglobin (Hb) and ions in the same samples. Water extracts were analyzed for both ion and Hb content. Intracellular K^+^, Na^+^ and Rb^+^ content was determined by flame emission on a Perkin-Elmer AA 306 spectrophotometer. The flame spectrophotometer was calibrated using solutions of 0–100 μM KCl or NaCl or 0–50 μM RbCl in distilled water. Rb^+^ was used as physiological analogue of K^+^ to assess the influx of K^+^ mediated by the Na/K pump. The concentration of ions in erythrocytes was calculated from the measured content of ions in mmol/g Hb, assuming an Hb content of ~340 g/l of cells and a cell water volume of 0.75 l/l of cells [3]. Hb was determined by its optical density (OD) at 540 nm on a Spekol-11 spectrophotometer (Carl Zeiss Jena, Germany) in the same samples in which K^+^, Na^+^, and Rb^+^ were measured.

U937 cells were pelleted in RPMI medium and washed 5 times with MgCl_2_ solution without resuspension. Ions were extracted by 5% TCA solution. TCA extracts were analyzed for ion content on a Perkin-Elmer AA 306 spectrophotometer with standard TCA solutions. The TCA precipitates were dissolved in 0.1 N NaOH and analyzed for protein by the Lowry procedure, with serum bovine albumin as a standard. Cell water content was determined by measurements of the buoyant density of the cells in continuous Percoll gradient as described in our previous study [12–15]. In our experience, measurement of buoyant density of cells is the most sensitive and reliable of all currently existing method for determination of cell water. A buoyant density difference of 0.005 g/ml corresponds to a change in the water content of the cells by about 10% and results in a displacement of the cells by about 1 cm in the density gradient. The relative changes in cell water do not noticeably depend on the accepted values of ρ_dry_ or the ratio of protein to dry mass, in contrast to the absolute values of the water content. The ion concentration in U937 cells was calculated from the measured ion content in mmol per 1 g of protein and the water content in ml per 1 g of protein.

Rb^+^ influx was determined by adding 2.5 mM RbCl for 1 h to the RBC or for 10 min to U937 cells suspension. Preliminary studies revealed that Rb^+^ influx in RBCs was linear up to 2 h. Rb^+^ influx via Na/K pump was evaluated from the difference between Rb^+^ uptake in the presence and absence of ouabain. The pump Na^+^ efflux was calculated from ouabain-sensitive (OS) Rb^+^ influx assuming proportions of [Rb]_o_ and [K]_o_ of 2.5 and 5.8 mM, respectively, and Na/K pump flux stoichiometry of 3:2.

### Statistical analysis

Results are presented as means ± SD. Due to variations in the data on erythrocytes from different donors, the figure and table show the results of n independent experiments for the same particular donor. In each case, similar results were obtained in two or more separate experiments with cells from different donors (not shown). Statistical analysis for calculated data is not applicable.

### Computation

The mathematical background of the modeling has been described in details earlier [9–14]. The mathematical model of the movement of monovalent ions across the cell membrane is like that used by Jakobsson [9] and Lew with colleagues [1,3]. The considered approach makes it possible to calculate the fluxes of major monovalent ions along all main pathways through the cell membrane: Na/K pump, electrically conductive channels, and cation–chloride cotransporters NC, KC, and NKCC. All cation-chloride cotransporters belong to the known family SLC12A carrying monovalent ions with stoichiometry 1Na^+^:1K^+^:2Cl‾ (NKCC) or 1K^+^:1Cl‾ (KC) or 1Na^+^:1Cl‾ (NC). The latter can be represented by a single protein, the thiazide-sensitive Na-Cl cotransporter (SLC12 family), or by coordinated operation of the exchangers Na/H, SLC9, and Cl/HCO_3_, SLC26 [38]. The movement of each ion in our mathematical description is determined thermodynamically, not by the molecular structure of the ion pathway, and is characterized by a single rate coefficient for each transporter type.

Basic symbols and definitions used are shown in **Table 1.** Two mandatory conditions of macroscopic electroneutrality and osmotic balance are:

$${\left[Na\right]}_{i}+{\left[K\right]}_{i}-{\left[Cl\right]}_{i}+\frac{zA}{V}=0$$ (1)

$${\left[Na\right]}_{i}+{\left[K\right]}_{i}+{\left[Cl\right]}_{i}+\frac{A}{V}={\left[Na\right]}_{o}+{\left[K\right]}_{o}+{\left[Cl\right]}_{o}+{\left[B\right]}_{o}$$ (2)

The flux equations:

$$\frac{dNa_{i}}{dt}=V\{(p_{Na}u({\left[Na\right]}_{i}\exp(u)-{\left[Na\right]}_{o})/g-\beta {\left[Na\right]}_{i}+J_{NC}+J_{NKCC}\}$$ (3)

$$\frac{dK_{i}}{dt}=V\{(p_{K}u({\left[K\right]}_{i}\exp(u)-{\left[K\right]}_{o})/g+\beta {\left[Na\right]}_{i}/\gamma +J_{NKCC}+J_{KC}\}$$ (4)

$$\frac{dCl_{i}}{dt}=V\{(p_{Cl}u({\left[Cl\right]}_{o}\exp(u)-{\left[Cl\right]}_{i})/g+J_{NC}+J_{KC}+2J_{NKCC}\}$$ (5)

**Table 1:** Symbols and definitions.

Symbols in software	Symbols in text	Definitions and units
Na, K, Cl	Na^+^, K^+^, Cl^–^, Rb^+^	Ion species
NC, NKCC, KC		Types of cation-chloride cotransporters
na, k, cl, na0, k0, cl0	[Na]_i_, [K]_i_, [Cl]_i_ [Na]_o_, [K]_o_, [Cl]_o_	Concentration of ions in cell water or external medium, mM
naC, kC, clC	Na_i_, K_i_, Cl_i_	Content of ions in cell per unit of *A*, mmol∙mol^-1^
B0	[B]_0_	External concentrations of membrane-impermeant non-electrolytes such as sucrose introduced sometimes in artificial media, mM
A	*A*	Intracellular content of membrane-impermeant osmolytes, mmol, may be related to g cell protein or cell number, etc.
V	*V*	Cell water volume, ml, may be related to g cell protein or cell number, etc.
A/V*1000		Membrane-impermeant osmolyte concentration in cell water, mM
V/A		Cell water content per unit of *A*, ml∙mmol^-1^
z	*z*	Mean valence of membrane-impermeant osmolytes *A*, dimensionless
pna, pk, pcl	pNa, pK, pCl; *p_Na_, p_K_, p_Cl_*	Permeability, rate coefficients, min^-1^
beta	*β*, *beta*	Pump rate coefficient, min^-1^
gamma	*γ*	Na/K pump flux stoichiometry, dimensionless
U	*U*	Membrane potential, MP, mV
	*u*	Dimensionless membrane potential *U* = *u*RT/F, dimensionless
NC, KC, NKCC	*J*_NC_, *J*_NKCC_, *J*_KC_	Net fluxes mediated by cotransport, μmol∙min^-1^∙ (ml cell water)^-1^
PUMP	*-β*[Na]_i_	Na efflux via the pump, μmol∙min^-1^∙ (ml cell water)^-1^
PUMP	*β*[Na]_i_/γ	K influx via the pump, μmol∙min^-1^∙ (ml cell water)^-1^
Channel		Net fluxes mediated by channels, μmol∙min^-1^∙ (ml cell water)^-1^
IChannel, INC, IKC, INKCC		Unidirectional influxes of Na, K or Cl via channels or cotransport, μmol∙min^-1^∙ (ml cell water)^-1^
EChannel, ENC, EKC, ENKCC		Unidirectional effluxes of Na, K, or Cl via channels, or cotransport, μmol∙min^-1^∙ (ml cell water)^-1^
inc, ikc	*inc*, *ikc*	NC, KC cotransport rate coefficients, ml∙μmol^-1^∙min^-1^
inkcc	*inkcc*	NKCC cotransport rate coefficients, ml^3^∙μmol^-3^∙min^-1^
kv		Ratio of “new” to “old” media osmolarity when the external osmolarity is changed, dimensionless
hp		Number of time points between output of results, dimensionless
mun, muk, mucl	*Δμ*_Na_, *Δμ*_K_, *Δμ*_Cl_	Transmembrane electrochemical potential difference for Na^+^, K^+^, or Cl^–^, mV
OSOR	OSOR	Ratio of ouabain-sensitive to ouabain-resistant Rb^+^ (K^+^) influx, dimensionless
kb		Parameter characterizing a linear decrease of the pump rate coefficient *β* with time, min^-1^

The left-hand sides of these flux equations represent the rates of change of cell ion content. The right-hand sides express fluxes, where *u* is the dimensionless term for membrane potential related to the its absolute values *U* (mV), as *U* = *u*RT/F = 26.7*u* for 37°C and *g* = 1-exp(*u*). The rate coefficients *p_Na_, p_K_, p_Cl_* characterizing channel ion transfer are similar to the Goldman’s coefficients. Fluxes *J_NC_, J_KC_, J_NKCC_* depend on internal and external ion concentrations as

$$J_{NC}=inc\cdot ({\left[\mathrm{Na}\right]}_{o}{\left[\mathrm{Cl}\right]}_{o}-{\left[\mathrm{Na}\right]}_{i}{\left[\mathrm{Cl}\right]}_{i})$$ (6)

$$J_{KC}=ikc\cdot ({\left[K\right]}_{o}{\left[\mathrm{Cl}\right]}_{o}-{\left[K\right]}_{i}{\left[\mathrm{Cl}\right]}_{i})$$ (7)

$$J_{NKCC}=inkcc.({\left[\mathrm{Na}\right]}_{o}{\left[K\right]}_{o}{\left[\mathrm{Cl}\right]}_{o}{\left[\mathrm{Cl}\right]}_{o}-{\left[\mathrm{Na}\right]}_{i}{\left[K\right]}_{i}{\left[\mathrm{Cl}\right]}_{i}{\left[\mathrm{Cl}\right]}_{i})$$ (8)

Here *inc*, *ikc*, and *inkcc* are the rate coefficients for cotransporters.

The input data (**S2 File**) are the following: extracellular and intracellular concentrations (*na0*, *k0*, *cl0* and *B0*; *na*, *k* and *cl*); *kv*; the pump rate coefficient (*β*); the pump Na/K stoichiometric coefficient (*γ*); parameter *kb*; channel permeability coefficients (*pna*, *pk*, *pcl*); and the rate coefficients for the NC, KC and NKCC cotransporters (*inc*, *ikc*, *inkcc*). The parameter *kv* is the ratio of the osmolarity of the external medium to the osmolarity of the intracellular one. The sodium pump rate coefficient (*β*) is defined as the ratio of the pumped flux of Na^+^ to [Na^+^] in the cell, assuming a linear dependence of the efflux of Na^+^ on intracellular [Na^+^] in the studied range of concentrations. The parameter *kb*, coefficient of a linear decrease of *beta* over time, is introduced to calculate the disturbance of cellular ion homeostasis, accompanied by a gradual decrease in the pump activity, as was the case with staurosporine-induced apoptosis of U937 cells in our previous study [12,13]. Such a case, when the parameter *kb* is nonzero, is not considered in the current study.

The results of computations appear in the file RESB.txt (an example of this is the "**S4 File**"). The algorithm and boundary conditions used in calculation are detailed in [10,11]. The solution of the system of equations is limited by physically significant values of the parameters or initial concentrations. It should be noted that 1:1 Na/Na and Cl/Cl exchange fluxes are omitted in the calculations by the executable file BEZ02BC. Their calculation is considered in our previous study [11].

The executable file of the BEZ02BC program used in this study with two auxiliary files is presented in the Supporting information. To use the executable file for the BEZ02BC software, you must open file **“S1 File”** and execute it according to its text. Other (new) version of the program adapted to display graphics is available on the website https://vereninov.com/cellionfluxes.

## Results

### Computation of ion flux balance in RBC

Using a model with single parameters for characterization of each ion pathways (Na/K pump, Na^+^, K^+^, and Cl^−^ channels, and NC, KC and NKCC cotransporters) is sufficient for successful description of the homeostasis in real animal cells at a real accuracy of the available experimental data [10–12]. Rate coefficients for each ion pathway can be calculated from known concentrations of monovalent ions and ouabain-sensitive and **-**resistant components of the Rb^+^(K^+^) influxes measured in cells under normal physiological conditions, and, if available, data of inhibitory analysis. Earlier, we obtained different sets of balance parameters for proliferating lymphoid U937 cells [13]. The found parameters provide entire ion and water homeostasis similar to that obtained in the experiment. The values of these parameters can vary depending on the physiological state of cells, cell culture age, etc. The left column of **Table 2** shows one of the set of standard parameters for U937 cells obtained earlier (cells A in [14]). **Table 2** also shows the parameter sets of 3 types of erythrocytes: donor RBC-1 (this study, except for [Cl]_i_, which is taken from a review by Balach et al. [39]; RBC-2 with ion concentrations taken from a review by Balach et al. [39] obtained experimentally by Funder and Wieth [17,18]; RBC-3 with “measured” characteristics, as used in the calculations by Lew et al. [1,3]. For different sets of initial "measured" parameters, the calculated parameters also differ somewhat. Comparison of the data calculated for different cells shows that the K^+^ and Na^+^ rate constants for erythrocytes are in all cases noticeably lower than for proliferating U937 cells, which is in accordance with a known low cation permeability of erythrocyte membranes [26,40].

**Table 2:** Basic characteristics of ion distribution measured and computed for U937 cells and 3 types of RBCs equilibrated with a standard medium, containing in mM: Na^+^ 140, K^+^ 5.8, Cl^-^ 116; pH 7.4.

Characteristics and parameters	U937 cells	RBC-1	RBC-2	RBC-3
Measured characteristics				
[K^+^]_i_, mM	156 ± 9	138 ± 9	133.7	140
[Na^+^]_i_, mM	35 ± 5	12 ± 1	16.5	10
[Cl^–^]_i_, mM	70 ± 8	75.7	75.7	95
*beta*	0.039 ± 0.006	0.003	0.002	0.004
OSOR	3.56 ± 0.68	1.29	0.83	2.0
Computed characteristics and parameters				
*U*, mV	-45.2	-11.6	-11.7	-5.7
pNa	0.0019	7E-5	7E-5	3E-5
pK	0.01	8E-5	8E-5	3.5E-5
pCl	0.004	0.01	0.01	0.01
inc	7E-5	1.4E-6	1.7E-6	2.45E-6
ikc	3E-5	1.8E-6	1.1E-6	1.65E-6
inkcc	8E-9	1.5E-9	2.3E-9	1.1E-9

Along with the data presented in Table 2, the computer program gives a complete list of net and unidirectional fluxes of monovalent ions. The unidirectional and total fluxes of K^+^ and Na^+^ through all considered pathways for RBC-1 are presented in **Table 3**. The first line for each ion shows the fluxes calculated for erythrocytes equilibrated with the standard medium (**Table 3**, lines t = 0). According to our calculations, the proportion of K^+^ fluxes through electrodiffusional channels in the entire balance of RBC at rest is small compared to normal pump flux and flux along the NKCC pathway.

**Table 3:** Dynamics of the net and unidirectional fluxes of K^+^ and Na^+^ after stopping the Na/K pump with ouabain, calculated at constant parameters as in RBC-1 equilibrated with the standard medium.

Ion	Time with Oua (min)	Net fluxes, total	Unidirectional fluxes								Net fluxes			
			Influxes				Effluxes							
			Pump	IChann	IKC	INKCC	Pump	EChann	EKC	ENKCC	Pump	Channel	KC	NKCC
K^+^	0	0	0.0234	0.0006	0.0012	0.0164	0	-0.0089	-0.0187	-0.0139	0.0234	-0.0083	-0.0175	0.0024
	100	-0.0267	0	0.0006	0.0012	0.0164	0	-0.0087	-0.0186	-0.0176	0	-0.0082	-0.0173	-0.0012
	600	-0.0339	0	0.0006	0.0012	0.0164	0	-0.0078	-0.0164	-0.0279	0	-0.0072	-0.0152	-0.0115
	1200	-0.0329	0	0.0006	0.0012	0.0164	0	-0.0067	-0.0136	‐0.0308	0	-0.0061	-0.0124	‐0.0144
	2400	-0.0242	0	0.0006	0.0012	0.0164	0	-0.0049	-0.0092	-0.0284	0	-0.0042	-0.0080	-0.0120
	3600	-0.0174	0	0.0007	0.0012	0.0164	0	-0.0035	-0.0065	-0.0256	0	-0.0029	-0.0053	-0.0092
			Pump	IChann	INC	INKCC	Pump	EChann	ENC	ENKCC	Pump	Channel	NC	NKCC
Na^+^	0	0.	0	0.0121	0.0224	0.0164	-0.0351	-0.0007	-0.0012	-0.0139	-0.0351	0.0114	0.0212	0.0024
	100	0.0310	0	0.0120	0.0227	0.0164	0	-0.0008	-0.0016	-0.0176	0	0.0111	0.0211	-0.0012
	600	0.0190	0	0.0121	0.0227	0.0164	0	-0.0015	-0.0029	-0.0279	0	0.0106	0.0199	-0.0115
	1200	0.0150	0	0.0126	0.0227	0.0164	0	-0.0021	-0.0038	‐0.0308	0	0.0105	0.0189	‐0.0144
	2400	0.0158	0	0.0134	0.0227	0.0164	0	-0.0031	-0.052	-0.0284	0	0.0103	0.0175	-0.0120
	3600	0.0166	0	0.0138	0.0227	0.0164	0	-0.0041	-0.0066	-0.0256	0	0.0097	0.0161	-0.0092

*Beta* is pump rate coefficient; OSOR—ratio of ouabain-sensitive to ouabain-resistant Rb^+^ (K^+^) influx; pNa, pK, pCl—channel permeability coefficients; inc, ikc, inkcc–cotransport rate coefficients. Data on U937 cells are taken from our earlier study [14] (cells A). RBC-1 [K^+^]_i_ and [Na^+^]_i_ are means ± SD of 4 independent experiments for one particular donor in this study; [Cl^-^]_i_ is taken from a review by Balach et al. [39]. Ion concentrations for RBC-2 were obtained experimentally by Funder and Wieth [17,18]. Measured characteristics of RBC-3 are taken as in the calculations of Lew et al. [1,3].

Fluxes are given in μmol min^-1^ (ml cell water)^-1^. Calculation was performed with the parameters of RBC-1 given in **Table 2** as described in section “Materials and Methods.” Initial beta 0.003 changes to 0 at t > 0. Extremum in the ENKCC and net NKCC fluxes is marked in yellow. Similar results were obtained for RBC-2 and RBC- 3.

### Changes in cell ionic homeostasis of erythrocytes caused by stopping the sodium pump with ouabain compared to U937 cells

**Fig 1:**
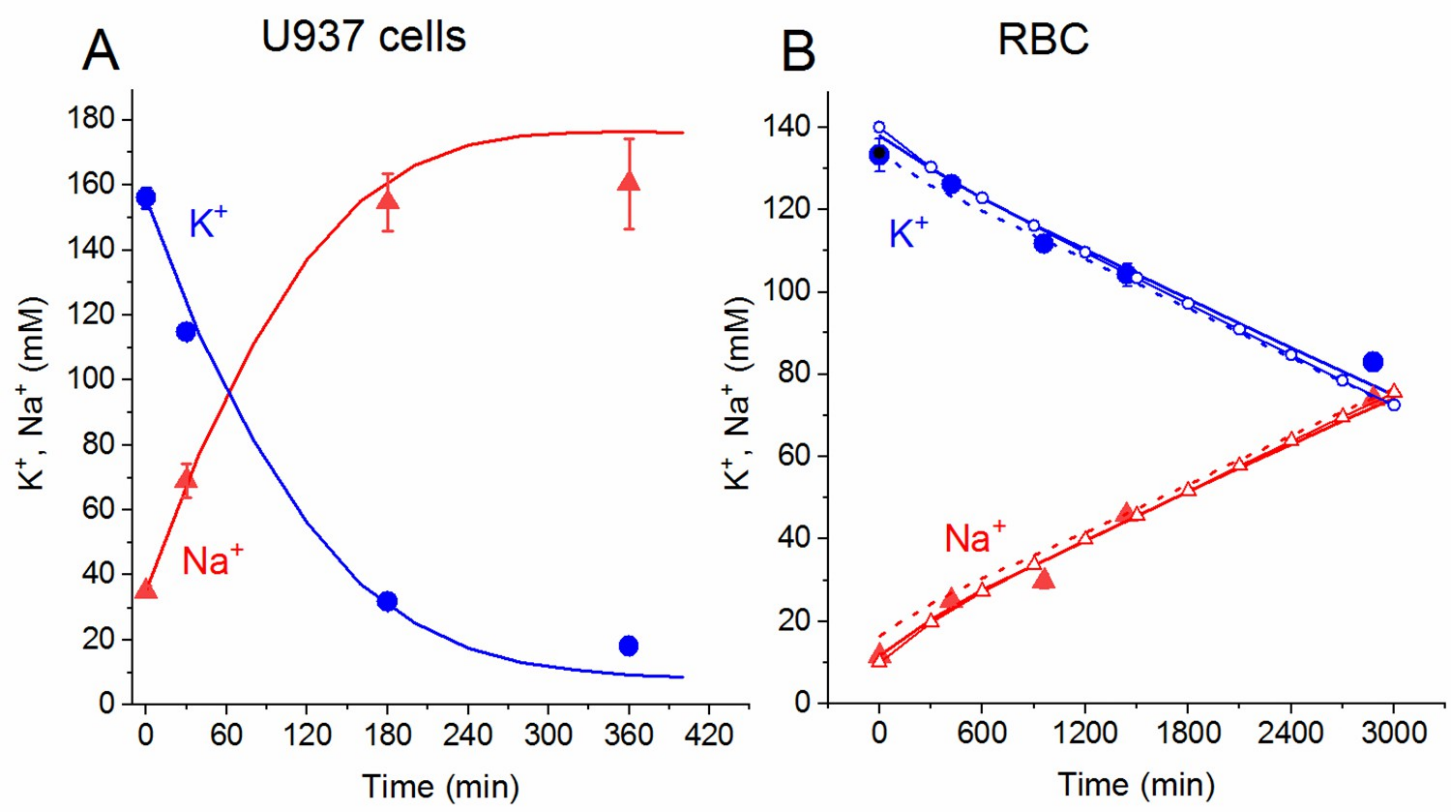
The time course of changes in K^+^ and Na^+^ concentrations in U937 cells (A) and in RBCs (B) after stopping the Na/K pump by ouabain. Large symbols show the experimental data. The lines show the calculated data obtained with the same parameters as in cells equilibrated with a standard medium. (A) Experimental data for U937 cells are taken from [14], Cells A, means ± SD for 4 experiments with duplicate determinations. Small SD values are masked by symbols. (B) Experimental data for RBC-1 are means ± SD for 2 experiments with duplicate determinations. Lines are calculated data for RBC-1 (solid lines), RBC-2 (dashed lines), and RBC-3 (lines with small symbols) obtained with the parameters given in **Table 2.** The pump was blocked by ouabain at t = 0, which corresponds to a change of *beta* to 0. Experimental data on the concentration of ions are calculated as described in Methods.

Previous studies have shown that the dynamics of real-time changes in the ion homeostasis of U937 cells after stopping the pump with ouabain is well predicted by calculations based on the simplest model including the Na/K pump, Na^+^, K^+^, and Cl^−^channels, and cotransporters NC, KC and NKCC, even if the calculations were carried out with invariable parameters of channels and cotransporters found for normal untreated cells [10,11,14]. Both experiments and mathematical modeling study of changes in ionic and water homeostasis in U937 cells shows that after blocking the pump with ouabain, the system comes to a new balanced state within several hours (**Fig 1A**). The dynamics of changes in the ion homeostasis of erythrocytes after stopping the pump is much slower compared with proliferating lymphoid cells due to their significantly slower ion traffic (**Fig 1B,** note the different scales along the time axis in A and B). It may take several days to achieve a new balance state of RBCs under these conditions.

The dynamics of changes in ionic concentrations for erythrocytes, calculated using the parameters given in **Table 2** (with the exception of the pump rate coefficient *beta* taken equal to 0) coincided well with the real one, as well as for U937 cells (**Fig 1A and 1B**). It is noteworthy that the ratio K/Na and the pump rate coefficient *beta* can differ in different samples (RBC-1, RBC-2, RBC-3 in **Table 2**), as well as the values of other parameters that ensure the balance of ion fluxes in a standard medium; nevertheless, in all cases, the calculated dynamics of changes in the ion homeostasis of erythrocytes, obtained with somewhat different initial values of the parameters, is similar (**Fig 1B**). It can be concluded that during the observed transient process after stopping the pump, when a cardinal shift of the ionic composition of cells occurs, the potassium concentration drops from 133 to 83 mM, and the sodium concentration increases from 12 to 74 mM, the parameters of the ion transport pathways through the RBC plasma membrane can remain unchanged, as was the case for U937 cells.

The calculated ion fluxes through different pathways after stopping the Na/K pump is shown in **Table 3**. According to the calculation even after 2.5 days of incubation with ouabain, it is still far from a new balance state with zero total net fluxes of K^+^ and Na^+^. It should be noted that blocking the pump with ouabain leads to a significant increase with an extremum in the efflux and net flux of K^+^ and Na^+^ through the NKCC (marked in yellow in **Table 3**). An extremum in ENKCC was also observed earlier in cells U937 (see Table 3 in [14]). It may be associated with a change in the electrochemical gradients of ions that determine the force driving the ion transport through the NKCC without changes in its regulation. It is noteworthy that the dynamics of cellular ion homeostasis after stopping the sodium pump with ouabain is well predicted by the proposed approach both in human erythrocytes and in U937 cells even without changes in the properties of membrane transporters and channels.

### Changes in the homeostasis of monovalent ions of erythrocytes in non-isoosmotic media. Comparison of real changes with calculated ones

**Fig 2:**
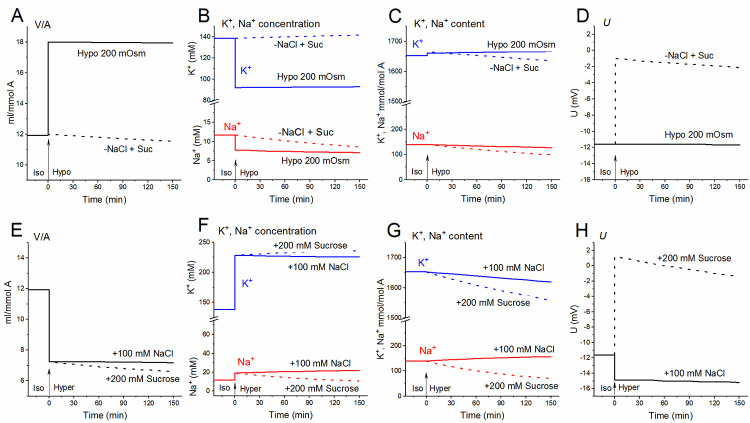
Calculated disturbance of ionic homeostasis in erythrocytes transferred to a hypotonic medium with a decrease in the concentration of NaCl (**A-D**) or to a hyperosmotic medium with the addition of NaCl or sucrose (**E-H**). **(A-D)** The standard medium was replaced at t = 0 with a hypotonic medium prepared by diluting the standard medium with distilled water (Hypo 200 mOsm, solid lines) or diluting with 300 mM sucrose (-NaCl + Suc, dashed lines) in a ratio of 2:1. **(E-H)** The standard medium was replaced at t = 0 with a hyperosmolar medium with 100 mM NaCl (solid lines) or 200 mM sucrose (dashed lines). The calculated data were obtained with RBC-1 parameters unchanged over time, shown in Table 2.

In view of the significant difference between the membrane permeability of U937 cells and RBCs, it was interesting to compare the time response of cells to osmotic challenge. Since water penetrates the cell membrane faster than osmolytes, after the transition of erythrocytes into a hypo- or hyperosmolar medium, the water content in the cell and the intracellular ion concentrations change sharply according to the well-known law of water-osmotic balance (**Fig 2**). We studied not only the effect of reducing the total osmolarity of the medium when the decrease in NaCl was achieved by diluting the medium with water, but also the effect of reducing NaCl without changing the total osmolarity when the standard medium was diluted with isoosmolar sucrose, to obtain a medium with low ionic strength (LIS) (**Fig 2A–2D**, dashed lines labeled “-NaCl + sucrose”). Thus, we have separated the effects of reducing the osmolarity of the medium and reducing the ionic strength. In the latter case, no abrupt changes in cell volume and ion concentration were observed when calculating with invariable parameters, as in erythrocytes balanced with a standard medium. Our calculations show an increase in the membrane potential of erythrocytes both in the LIS medium and in the hypertonic medium with sucrose (**Fig 2D** and **2H**). Such changes in the membrane potential are due to changes in the electrochemical gradients of ions, i.e. the force driving ions through the plasma membrane. Predicted changes are similar to observed by Bisognano, who studied the effect of hypertonic sucrose on human erythrocytes [41]. The effect of LIS medium on changes in erythrocyte homeostasis was also discussed in a study by Moersdorf et al. [42]. The authors note a large increase in the membrane potential when the RBCs were transferred into an isosmotic LIS medium containing sucrose. According to our calculations, this increase does not exceed 10 mV. Unfortunately, measurement of fast changes of membrane potential is difficult task and connected with use of special methods of correction [41].

A detailed analysis of changes in the ionic and water balance of U937 cells under osmotic challenge was carried out in our previous studies [14,15]. It was shown that the well-known cellular reactions of RVD (Regulatory Volume Decrease) and RVI (Regulatory Volume Increase), which underlie the adaptation of animal cells to a hypo- or hyperosmolar environment, can occur in cells like U937 without any specific “regulatory” changes in the properties of membrane transporters and channels, only due to changes in electrochemical ionic gradients. Over time, in U937 cells placed in a hypo- or hyperosmolar medium, a new equilibrium distribution of monovalent ions was established. Unlike U937 cells, erythrocytes do not show visible RVD or RVI reactions, as shown by the calculations with unchanged parameters, as in cells equilibrated with a standard medium of 310 mOsm (**Fig 2A and 2E**).

**Fig 3:**
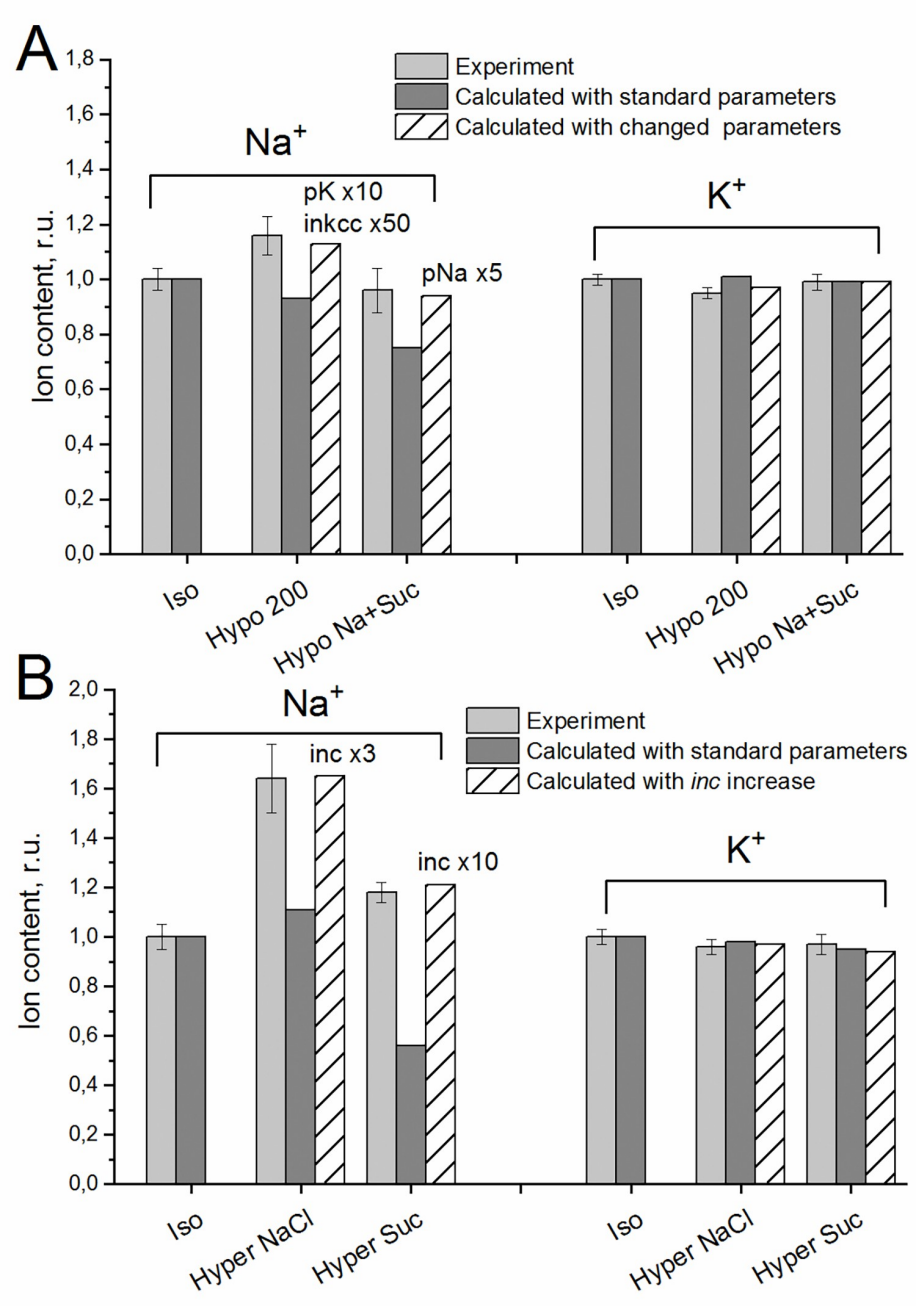
Comparison of experimental and calculated data on changes in the content of Na^+^ and K^+^ in erythrocytes incubated in media with a low content of NaCl (**A**) or in hyperosmolar media with the addition of NaCl or sucrose (**B**) for 2 hours. The data are presented in relative units; the ion content in a standard medium of 310 mOsm (Iso) is taken as 1. Data in absolute units are given in S1 Table. Experimental data are means ± SD of 2–3 independent experiment with duplicate determination. The calculated data were obtained either with the standard parameters of RBC-1 given in Table 2 (parameters unchanged), or when changing the parameters shown in the graph above the columns. Parameters for hypoosmolar medium: na0 90, k0 4, cl0 75, B0 31, kv 0.645. Parameters for hyperosmolar medium with NaCl: na0 240, k0 5.8, cl0 216, B0 48.2, kv 1.645; or with sucrose: na0 140, k0 5.8, cl0 116, B0 248.2, kv 1.645.

Comparison of real changes in the K^+^ and Na^+^ content of erythrocytes with calculated ones, when they are caused by a change in the osmolarity of the medium, is shown in **Fig 3 and S1 Table**. After incubation in a hypoosmolar medium for 2 h (Hypo 200 in **Fig 3A**), the Na^+^ content in real cells increases, while the calculation with unchanged parameters gives a slight decrease in the Na^+^ content. When the standard medium is diluted with an isotonic sucrose solution (Hypo Na+Suc in **Fig 3A**), the Na^+^ content in the real cells practically does not change, while, according to the calculation it decreases. After incubation of erythrocytes for 2 h in a hyperosmolar medium with NaCl (Hyper NaCl in **Fig 3B**), an increase in the Na^+^ content was observed which in real cells was noticeably higher than in the calculation. In a hyperosmolar medium with the addition of sucrose (Hyper Suc in **Fig 3B**), the Na^+^ content according to the calculation decreases, while in the experiment it slightly increases. The content of K^+^ in all these cases changes less significantly both in the experiment and in the calculations.

These discrepancies between experimental data and calculations performed using standard parameters indicate that when cells are transferred to a hypo- or hyperosmolar medium, the parameters of the ion transport pathways through the cell membrane change. Some parameters need to be changed to match the experimental and calculated data. Namely, the simulation done with increased pK and *inkcc* shows the increase in Na^+^ content in hypoosmolar medium, similar to observed in experiment (**Fig 3A**). For cells placed in a medium with a low content of NaCl and with the addition of sucrose, the calculation shows that there will be no decrease in the content of Na^+^ with an increase in pNa (**Fig 3A**). In a hyperosmolar medium, the calculated data will coincide with the experimental ones with an increase in the NC rate coefficient (**Fig 3B**). However, the available data are insufficient to further differentiate the mechanism of erythrocyte response in these cases.

The above data show how calculations can be used to find out what changes in the properties of ion transport pathways across the membrane can be responsible for the observed change in ion homeostasis. The effect of the parameters of ion transport pathways through the cell membrane on the ionic and water balance of the cell was analyzed in detail in our previous studies on the example of U937 cells [14,15]. The results of these studies show the importance of modeling for understanding the mechanisms of cell functioning.

## Discussion

Many different processes are involved in the regulation of the functioning of a living cell. Due to their interrelations, it is difficult or even impossible to isolate one or another specific mechanism. The solution of many problems in this area cannot be solved currently without computer simulation. Despite the great achievements of molecular biology, it should be not forgotten about the fundamental physico-chemical regularities of cell functioning. We have recently developed software based on the concept of the cell as an electrochemical system that allows us to calculate the balance of transmembrane fluxes of K^+^, Na^+^ and Cl^−^through main ion transport pathways. It is known that ion transport plays an important role in the regulation of the ion-water balance and the difference in electrical potentials between the cell and the environment. The calculation of the balance of ion fluxes will make it possible to analyze the mechanisms of ion transport through the cell membrane.

The proposed software is quite simple and convenient even for users who are not familiar with programming. The system of equations, all used parameters and the calculation algorithm are described in detail earlier in [10,11] and presented in this article partially. Previously, the usefulness of our approach was clearly demonstrated by studying the mechanism of the early stages of staurosporine-induced apoptosis in U937 cells [12]. Using a computational tool, we showed that the alteration in the ionic and water balance in apoptotic U937 cells is associated with a decrease in the Na/K pump rate coefficient and a change in the permeability of K^+^, Na^+^, and Cl^-^ channels, which was in full agreement with the literature data. Next, we performed a detailed analysis of the response of cells like U937 to changes in the osmolarity of the external environment, RVD and RVI [14,15]. Comparison of the calculated data with the experiment showed that the reaction of living cells is much more complicated than the calculation can predict. Nevertheless, the program allows you to determine what change in the ionic and water balance of the cell can be expected with a change in a particular parameter of the ion transport pathway.

In this paper, we present the results of testing our software on human erythrocytes, which differ significantly from U937 cells in term of functional and structural characteristics. The calculations are carried out taking into account the main pathways of ion movement through the cell membrane: the Na/K pump, K^+^, Na^+^, Cl^-^ channels, and NC, KC, and NKCC cotransporters. All these cotransporters were included in the calculation of unidirectional fluxes of monovalent ions in RBC, all of them are present in human erythrocytes and their role in the functioning of erythrocytes can be considered known (see reviews [25,26]). In normal mature erythrocytes, KC cotransport is largely quiescent, but can be activated under certain conditions [43,44]; its activity is abnormally elevated in red blood cells from patients with sickle cell disease [28]. NKCC cotransporter in normal human erythrocytes shows tenfold inter-individual variation between different donors and is related to cell water сontent [22]. Cell shrinkage is known to increase and cell swelling to decrease NKCC cotransport in human erythrocytes [45].

Cationic homeostasis in RBC is maintained mainly by the active movement of Na^+^ and K^+^ through the Na/K pump, coupled with relatively lower passive permeability through other transport pathways. Our calculations showed that the experimentally obtained characteristics of erythrocytes, given in **Table 2,** can be realized only at low values of the permeability of K^+^ and Na^+^ channels and at more than 2 orders of magnitude higher permeability of Cl^-^ channels, which is consistent with the literature data [1].

The calculation of changes in the ion balance of erythrocytes after stopping the Na/K pump predicted well the experimentally obtained changes in the concentration of K^+^ and Na^+^ in cells treated with ouabain, as was the case in proliferating U937 lymphoid cells. It is noteworthy that in both cases this occurred without changing the properties of channels and transporters, i.e., without changing the transport rate constants. In full accordance with predictions the transient processes in proliferating lymphoid cells are much faster (hours) than in human erhytrocytes (days). Thus, the proposed approach has confirmed its reliability and can be used to calculate the balance of ion fluxes and their changes in erythrocytes under various conditions. The discrepancy between the measured data and calculations performed with unchanged standard parameters points to changes in the properties of the ion transport pathways. Identification of parameters, the change of which will lead to the coincidence of experimental and calculated data will help to outline the ways for further experimental research.

In some diseases, notably sickle cell disease, there is an increase in the cation permeability of the erythrocyte membrane, leading to the loss of cations and cell shrinkage. Recent studies provide more information on the molecular basis of membrane permeability enhancement. It is shown that increased cation permeability of red blood cells is provided mainly by activation of K^+^-selective channel (KCNN4, Gardos channel) and KC transporter [28,40]. Our mathematical description is not related to the molecular basis of ion transport pathways. All transport pathways are divided according to the thermodynamics (by the ion-driving force) into 3 types: channels (K^+^, Na^+^, Cl^-^), where the driving force is the transmembrane electrochemical potential difference for a single ion species, cotransporters (NC, KC, and NKCC), where the driving force is the sum of the electrochemical potential differences for all partners, and the Na/K ATPase pump, where ion movement against electrochemical gradient is energized by ATP hydrolysis. A single integral permeability coefficient for each ion and each pathway through the plasma is used in the calculation of the balance of monovalent ion fluxes.

The calculation allows one to see the relationship between ion fluxes through different pathways and to analyze how the movement of one ion through one of the transport pathways affects the fluxes of other ions. According to our calculations, the contribution of K^+^ fluxes through electrodiffusional channels in the entire balance of RBCs at rest and after stopping the sodium pump is small compared to normal pump flux and flux along the NKCC pathway (**Table 3**). This conclusion is consistent with the literature that calcium-activated potassium channels, KCNN4 or Gardos channels, are inactive under physiological conditions, usually exist in a dormant closed state and are not thought to play a major role within homeostatic physiological conditions (see reviews [26,40,46]). Most likely, Gardos channels, as well as the recently discovered mechanosensitive cationic PIEZO1 channels, which are inactive at rest, play the significant effecter and regulatory role, participating in specific physiological reactions of erythrocytes, for example, when passing through the capillary network [2,47]. Furthermore, the activity of the channels can change considerably under various pathological conditions [26,28,40,46,48].

Recently, we have studied the role of cation-chloride cotransporters, the Na/K pump, and channels in the regulation of cell water and ions in the response of U937 cells to a hypo- or hyperosmolar medium, both *in silico* and experimentally [14,15]. Computer analysis showed that RVD and RVI can occur in U937 cells without any changes in the properties of membrane transporters and channels due to time-dependent changes in electrochemical ion gradients. It was also shown how changes in the properties of membrane transporters and channels can affect the response of cells to changes in the medium osmolarity. Human erythrocytes exhibit very limited volume regulation capacity. While the response of U937 cells to hypertonic shrinkage or hypotonic swelling is very rapid (minutes), for erythrocytes these responses can be much slower. There are indications that human erythrocytes are among the very few cell types that lack volume regulatory mechanisms such as RVI or RVD [24,33,34]. Experimental studies of the response of the human erythrocytes to hypotonic swelling have been contradictory [22]. It is noted that volumetric-regulatory reactions of erythrocytes may be at the limit of experimental detection.

Our data on changes in the content of K^+^ and Na^+^ in cells with a change in the osmolarity of the medium show the discrepancy between the calculations carried out using the parameters obtained for cells equilibrated with a standard medium and the experimental data (**Fig 3).** This indicates that when cells are transferred to a non-isotonic medium, the parameters of the ion transport pathways through the cell membrane change. Previously, we have shown that an increase in the rate coefficients pK, pCl, and *ikc* enhances RVD in cells like U937 placed in a hypoosmolar medium [14], while an increase in *inc* increases RVI in a hyperosmolar medium [15]. In the sucrose hyperosmolar medium, RVI required a much greater increase in *inc* than in the NaCl hyperosmolar medium. This study showed that the response of living cells to osmotic challenge is more complicated than the electrochemical model predicts. The present study demonstrates that the experimentally observed increase in the content of Na^+^ in erythrocytes placed in a hypoosmolar medium can be obtained by increasing pK and *inkcc*, while in a hyperosmolar medium, the calculated data will coincide with the experimental ones with an increase in the NC rate coefficient. In a hyperosmolar medium with sucrose, a larger increase in *inc* is required to match the experimental data than in a hyperosmolar medium with NaCl. Thus, when the tonicity of the environment changes, both K^+^ channels and ion cotransporters, such as NC and NKCC, can participate in the regulation of ion homeostasis of human erythrocytes. A detailed consideration of what changes in the parameters can provide agreement between the calculated and experimental data is beyond the scope of this study and may be the subject of further research.

## Conclusion

The proposed approach, based on the use of the thermodynamic classification of ion transport systems across the cell membrane, including all types of cation-chloride cotransporters and independent of the mechanism of ion movement, makes it possible to calculate the balance of unidirectional fluxes of monovalent ions along all significant pathways, which seems to be very important in the study of functional expression of ion channels and transporters. The main result of this study is the successful testing of the developed computer program for describing homeostasis in human erythrocytes, which demonstrated the possibility of using our approach to assess changes in ion homeostasis not only of proliferating lymphoid cells but also of quiescent red blood cells.

Using the simple mathematical description of the monovalent ion fluxes via major pathways in human erythrocytes with only single rate coefficient for each ion and each pathway obtained for resting state allows predicting quantitatively transient processes in changing flux balance caused by stopping the sodium pump with ouabain. In full accordance with predictions the transient processes in proliferating lymphoid cells are much faster (hours) than in human erythrocytes (days).

The contribution of the potassium fluxes through electrodiffusional channels (such as Gardos channels, Piezo-channels, etc.) in the entire balance at rest and after stopping the sodium pump is, according to our calculations, small compared to normal pump flux and flux along the NKCC pathway. Comparison of real changes in the homeostasis of monovalent erythrocyte ions with calculated ones, when they are caused by reasons other than stopping the pump, allows us to find out which of the pathways can be responsible for pathway-specific changes in ion homeostasis.

## Supporting information

S1 Table Changes in the content of K^+^ and Na^+^ in erythrocytes incubated in media with a reduced content of NaCl or in hyperosmolar media with the addition of NaCl or sucrose for 2 hours. Comparison of experimental data with calculated ones.
(DOC)

S1 File How to use BEZ02BC. (DOC)

S2 File DATAB. (TXT)

S3 File BEZ02BC. (TXT)

S4 File RESB control. (TXT)

S5 File (DOC)
