# Supplementary material for: Unidirectional fluxes of monovalent ions in human erythrocytes compared with lymphoid U937 cells: Transient processes after stopping the sodium pump and in response to osmotic challenge
Source: PLoS One. 2023 May 4;18(5):e0285185. doi: 10.1371/journal.pone.0285185 (PMC10159352; doi:10.1371/journal.pone.0285185)
Supplement: S1 Table — Comparison of experimental data with calculated ones. (DOC) [file pone.0285185.s001.doc]

**S1 Table.** Changes in the content of K^+^ and Na^+^ in erythrocytes incubated in media with a reduced content of NaCl or in hyperosmolar media with the addition of NaCl or sucrose for 2 hours. Comparison of experimental data with calculated ones.

| Medium | Experimental  data | | Calculated with standard parameters | | Calculated with changed parameters | | Changed parameters |
| --- | --- | --- | --- | --- | --- | --- | --- |
|  | K^+^ | Na^+^ | K^+^ | Na^+^ | K^+^ | Na^+^ |
|  | mmol/g Hb | | mmol/mol A | | | |  |
| Standard | 310 ± 6 | 25 ± 1 | 1653 | 140 |  |  |  |
| Hypo 200 mOsm | 296 ± 6 | 29 ± 2 | 1665 | 130 | 1605 | 158 | pK x10, inkcc x50 |
| pHypo -NaCl + Sucrose | 307 ± 9 | 24 ± 2 | 1641 | 105 | 1642 | 131 | pNa x5 |
|  |  |  |  |  |  |  |  |
| Standard | 316 ± 9 | 22 ± 1 | 1653 | 140 |  |  |  |
| Hyper +100 mM NaCl | 304 ± 8 | 36 ± 5 | 1626 | 155 | 1608 | 231 | inc x3 |
| Hyper + 200 mM sucrose | 308 ± 13 | 26 ± 1 | 1574 | 78 | 1553 | 170 | inc x10 |

Experimental data are means ± SD of 2-3 independent experiment with duplicate determination. Calculated data are the content of ions in relation to the content of intracellular impermeant osmolytes (mmol/mol A). The calculation was carried out either with the standard RBC-1 parameters given in Table 2, or when changing the parameters indicated in the last column. For hypoosmolar medium parameters are: na0 90, k0 4, cl0 75, B0 31, kv 0.645. For hyperosmolar medium with NaCl parameters are: na0 240, k0 5.8, cl0 216, B0 48.2, kv 1.645; with sucrose are: na0 140, k0 5.8, cl0 116, B0 248.2, kv 1.645.
