# Supplementary material for: Unidirectional fluxes of monovalent ions in human erythrocytes compared with lymphoid U937 cells: Transient processes after stopping the sodium pump and in response to osmotic challenge
Source: PLoS One. 2023 May 4;18(5):e0285185. doi: 10.1371/journal.pone.0285185 (PMC10159352; doi:10.1371/journal.pone.0285185)
Supplement: S1 File — (DOC) [file pone.0285185.s002.DOC]

How to use the executable file for the program BEZ02BC.

1. BEZ02BC should be run on a 32-bit Windows OS

a. All three files (input data file **S2 DATAB.txt**, executable **S3 BEZ02BC.txt** and output file **S4 RESB control.txt**) should be located in the same folder.

b. Rename the **S2 DATAB.txt** and **S3 BEZ02BC.txt** files to **DATAB.txt** and **BEZ02BC.txt** respectively. Change the extension of BEZ02BC from .txt to .exe (do not try to open BEZ02BC.txt. It is unreadable!).

c. Run the executable file and wait until the process is completed.

d. The resulting output should be the same as in the file RESB control.txt.

e. Now you can model the processes by varying parameters in DATAB.txt. Check the DATAB, the file must correspond to the selected parameters and concentrations, click "Save" (simple “DATAB.txt” without specific name). Instead of zero values for concentrations, small non-zero values should be used (e.g., 0.001 mM).

f. Run the executable file BEZ02BC with appropriate extension and wait until the process is completed and the file RESB.txt appears. Rename and save the obtained file RESB.txt because in a new running cycle it will be lost. RESB files can be easily imported by ORIGIN or another program for further processing (the asterisks must be retained).

g. The displayed values of fluxes as well as OSOR correspond to the latest time point. The values of fluxes for other moments can be obtained by setting the necessary time interval with the *hp* value. It is necessary to perform several calculation cycles with a series of the corresponding *hp* to obtain the time course of the fluxes.

h. Some readers of our previous publications have expressed doubt that using our tool it is possible to obtain a unique set of parameters that provide an agreement between experimental and calculated data. Our mathematical comments on this matter can be found in Yurinskaya et al., 2019, P.12.

2. To run the executable file on a 64-bit machine, the following additional steps should be taken:

a. Download the School Pak package via Internet and run Norton Commander (NCD).

b. Set in NCD the same folder as the folder in Windows where the DATAB.txt and executable file BEZ02BC are located.

c. Correct the file DATAB.txt if necessary, in the Windows folder.

d. Run executable file in the NCD folder and read RESB.txt in the analogous Windows folder.

Several DATAB and appropriate RESB options are presented below as examples:

Example 1 , U937 cells:

DATAB

na0 k0 cl0 B0 kv na k cl beta gamma

140.0 5.8 116.0 48.2 1.0 35.0 156.0 70.0 0.039 1.50

pna pk pcl inc ikc inkcc hp kb

0.00190 0.010 0.004 0.000070 0.0000300 0.0000000080 300 0.0

RESB

t U na k cl V/A mun muk mucl naC kC clC

0 -45.2 35.0 156.0 70.0 20.41 -82.2 42.7 31.7 714.3 3183.7 1428.6

30 -45.2 35.0 156.0 70.0 20.41 -82.2 42.7 31.7 714.3 3184.1 1429.0

....................................................................................

300 -45.2 35.0 156.0 70.0 20.42 -82.2 42.7 31.7 714.5 3185.1 1430.2

na0 k0 cl0 B0 kv na k cl beta gamma

* 140.0 5.8 116.0 48.2 1.000 35.0 156.0 70.0 0.039 1.50

* pna pk pcl inc ikc inkcc hp kb

* 0.00190 0.01000 0.00400 0.0000700 0.0000300 0.0000000080 300 0.000000

Net_flux PUMP Channel NC KC NKCC

* Na -1.3647 0.5263 0.9652 0.0000 -0.1268

* K 0.9098 -0.4754 0.0000 -0.3076 -0.1268

* Cl 0.0000 -0.4039 0.9652 -0.3076 -0.2537

* Influx PUMP IChannel INC IKC INKCC

* Na 0.0000 0.5517 1.1368 0.0000 0.0874

* K 0.9098 0.1203 0.0000 0.0202 0.0874

* Cl 0.0000 0.1772 1.1368 0.0202 0.1748

* Efflux PUMP EChannel ENC EKC ENKCC

* Na -1.3647 -0.0254 -0.1716 0.0000 -0.2142

* K 0.0000 -0.5956 0.0000 -0.3278 -0.2142

* Cl 0.0000 -0.5811 -0.1716 -0.3278 -0.4285

* z OSOR (A/V)*1000

* -2.47 3.99 48.97

Example 2, Red Blood Cells-1

DATAB

na0 k0 cl0 B0 kv na k cl beta gamma

140.0 5.8 116.0 48.2 1.0 11.7 138.7 75.7 0.003 1.50

pna pk pcl inc ikc inkcc hp kb

0.00007 0.00008 0.010 0.0000014 0.0000018 0.0000000015 300 0.0

RESB

t U na k cl V/A mun muk mucl naC kC clC

0 -11.6 11.7 138.7 75.7 11.92 -77.9 73.1 0.2 139.5 1653.2 902.3

30 -11.6 11.7 138.7 75.7 11.92 -77.9 73.1 0.2 139.5 1653.1 902.3

60 -11.6 11.7 138.7 75.7 11.92 -77.9 73.1 0.2 139.6 1653.1 902.4

………………………………………………………………………………………………………………

240 -11.6 11.7 138.7 75.7 11.92 -77.8 73.1 0.2 139.9 1652.9 902.5

270 -11.6 11.7 138.7 75.7 11.92 -77.8 73.1 0.2 140.0 1652.8 902.5

300 -11.6 11.7 138.7 75.7 11.92 -77.8 73.1 0.2 140.0 1652.8 902.5

* na0 k0 cl0 B0 kv na k cl beta gamma

* 140.0 5.8 116.0 48.2 1.000 11.7 138.7 75.7 0.003 1.50

* pna pk pcl inc ikc inkcc hp kb

* 0.00007 0.00008 0.01000 0.0000014 0.0000018 0.0000000015 300 0.000000

* Net_flux PUMP Channel NC KC NKCC

* Na -0.0352 0.0114 0.0215 0.0000 0.0024

* K 0.0235 -0.0083 0.0000 -0.0177 0.0024

* Cl 0.0000 -0.0086 0.0215 -0.0177 0.0048

* Influx PUMP IChannel INC IKC INKCC

* Na 0.0000 0.0121 0.0227 0.0000 0.0164

* K 0.0235 0.0006 0.0000 0.0012 0.0164

* Cl 0.0000 0.9255 0.0227 0.0012 0.0328

* Efflux PUMP EChannel ENC EKC ENKCC

* Na -0.0352 -0.0007 -0.0012 0.0000 -0.0140

* K 0.0000 -0.0088 0.0000 -0.0189 -0.0140

* Cl 0.0000 -0.9341 -0.0012 -0.0189 -0.0280

* z OSOR (A/V)*1000

* -0.89 1.29 83.89
