# Supplementary material for: Unidirectional fluxes of monovalent ions in human erythrocytes compared with lymphoid U937 cells: Transient processes after stopping the sodium pump and in response to osmotic challenge
Source: PLoS One. 2023 May 4;18(5):e0285185. doi: 10.1371/journal.pone.0285185 (PMC10159352; doi:10.1371/journal.pone.0285185)
Supplement: S5 File — (DOC) [file pone.0285185.s006.doc]

A version of the **BEZ02BC** software adapted to display graphics is available at **https://vereninov.com/cellionflux**.
